# Retracted: Ginseng on Hyperglycemia: Effects and Mechanisms

**DOI:** 10.1155/2020/1698627

**Published:** 2020-11-24

**Authors:** Evidence-Based Complementary and Alternative Medicine

*Evidence-Based Complementary and Alternative Medicine* has retracted the article titled “Ginseng on Hyperglycemia: Effects and Mechanisms” [1], this article has been retracted as it is essentially identical in content with a previously published paper:

Wu Z, Luo JZ, Luo L. “American ginseng modulates pancreatic beta cell activities.” Chin Med. 2007 Oct 25; 2:11.

## References

[1] Luo J. Z., Luo L. (2009). Ginseng on Hyperglycemia: Effects and Mechanisms. *Evidence-Based Complementary and Alternative Medicine*.

